# Mutations in ALK and TSC1 in a gastrointestinal stromal tumor: a case report

**DOI:** 10.1186/s12893-021-01208-0

**Published:** 2021-04-20

**Authors:** Qingzhi Song, Guan Li, Zhuofei Li, Sheng Ao, Jianing Hou, Guoqing Lv

**Affiliations:** 1 Peking University Shenzhen Hospital, Clinical College of Anhui Medical University, Shenzhen, 518036 Guangdong People’s Republic of China; 2 Department of Gastrointestinal Surgery, Peking University Shenzhen Hospital, Shenzhen, 518036 Guangdong People’s Republic of China

**Keywords:** Gastrointestinal stromal tumor, Mutations, ALK, TSC1

## Abstract

**Background:**

Gastrointestinal stromal tumors rarely occur in children, but when they do, their biological behavior and histopathological patterns differ from those of adults.

**Case presentation:**

A 13-year-old boy with a gastrointestinal stromal tumor was characterized by a rare genetic mutation. The patient complained of “fatigue with intermittent abdominal pain for 1 month”. According to the preoperative imaging examination, gastroscopy, and gastroscopic biopsy, the patient was diagnosed with a gastric stromal tumor. Postoperative pathology showed that the tumor cells were fusiform and ovoid, and mitotic figures were easily seen. Immunohistochemistry revealed that the tumor was S-100(+), SOX10(−), CD34(+), SMA(partially+), DOG-1(+), CD117(+), KI-67 (positive for 20% + of the subjects and 40% + of the hotspots), and SDHB(−). Genetic tests showed missense mutations in ALK and TSC1. With surgical treatment, the tumor was completely removed. The patient recovered well and was discharged on the ninth day after the operation. He is currently under follow-up.

**Conclusions:**

In this case involving a patient with a gastrointestinal stromal tumor, immunohistochemistry indicated that the tumor was an "SDH-deficient type", and gene detection showed no KIT or PDGFRA mutation but rare ALK and TSC1 mutations, which adds to the knowledge of the types of gene mutations in children with gastrointestinal stromal tumors.

**Supplementary Information:**

The online version contains supplementary material available at 10.1186/s12893-021-01208-0.

## Background

A gastrointestinal stromal tumor (GIST) is the most common mesenchymal tumor in the gastrointestinal tract [1]. GISTs are primarily recognized by the expression of KIT proteins, and patients often have mutations in the KIT or platelet-derived growth factor receptor gene. GISTs rarely occur in children, and even then, they rarely present with mutations in KIT and PDGFRA, which we call wild-type GIST. Here, we describe a child with a GIST presenting with an SDH defect and a rare genetic mutation [2].

## Case presentation

A 13-year-old boy patient was admitted for "fatigue with intermittent abdominal pain for 1 month". In the prior month, the patient presented with fatigue symptoms without obvious incentives. However, intermittent paroxysmal abdominal pain and dizziness occurred after exercise, without abdominal distension, nausea, vomiting, acid reflux, and belching. The symptoms had no obvious periodicity or regularity. Before admission to our hospital, a routine blood examination in another hospital revealed a hemoglobin of 63 g/L, and abdominal enhanced CT showed gastric antrum surrounding a mass, thought to be either a stromal tumor or a lymphoma. The patient was physically fit, without any history of disease and surgery. No family history of illness. Physical examination on admission revealed the appearance of anemia, a flat abdomen, no varicose abdominal wall, no peristaltic waves, and no masses; the abdominal wall was soft, with no tenderness, rebound pain, or muscle tension. The liver, spleen, and subcostal areas were not palpable; no abnormal masses were palpated in the abdomen. Anemic tremors and water sounds were observed. There was no percussive pain in the liver and kidney areas. The number of bowel sounds is 4 per minute.

**Fig. 1:**
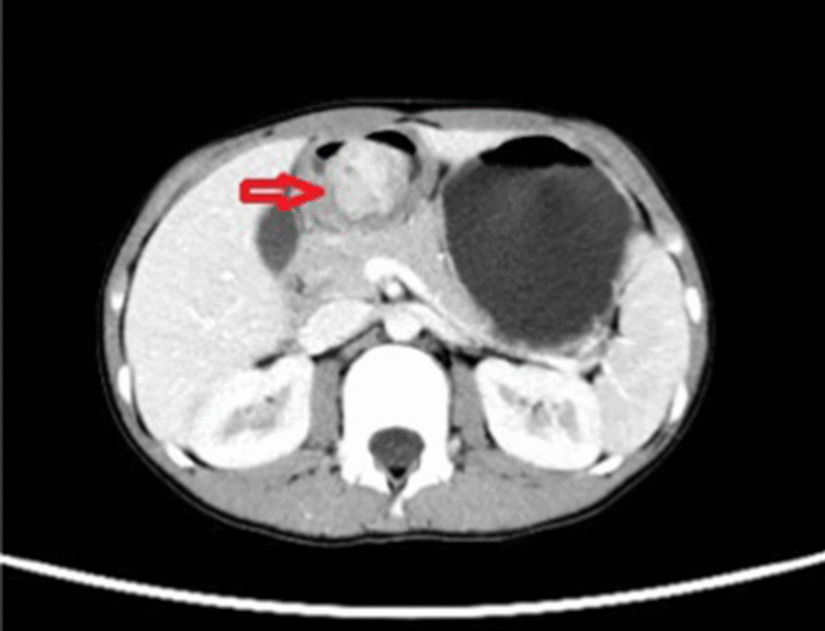
Computed tomography scan of the abdomen: in the arterial phase, a group of circular nonuniform enhanced shadows (red arrow) can be seen in the antrum cavity. The edges are poorly defined, and the size is approximately 40 × 33 mm^2^

**Fig. 2:**
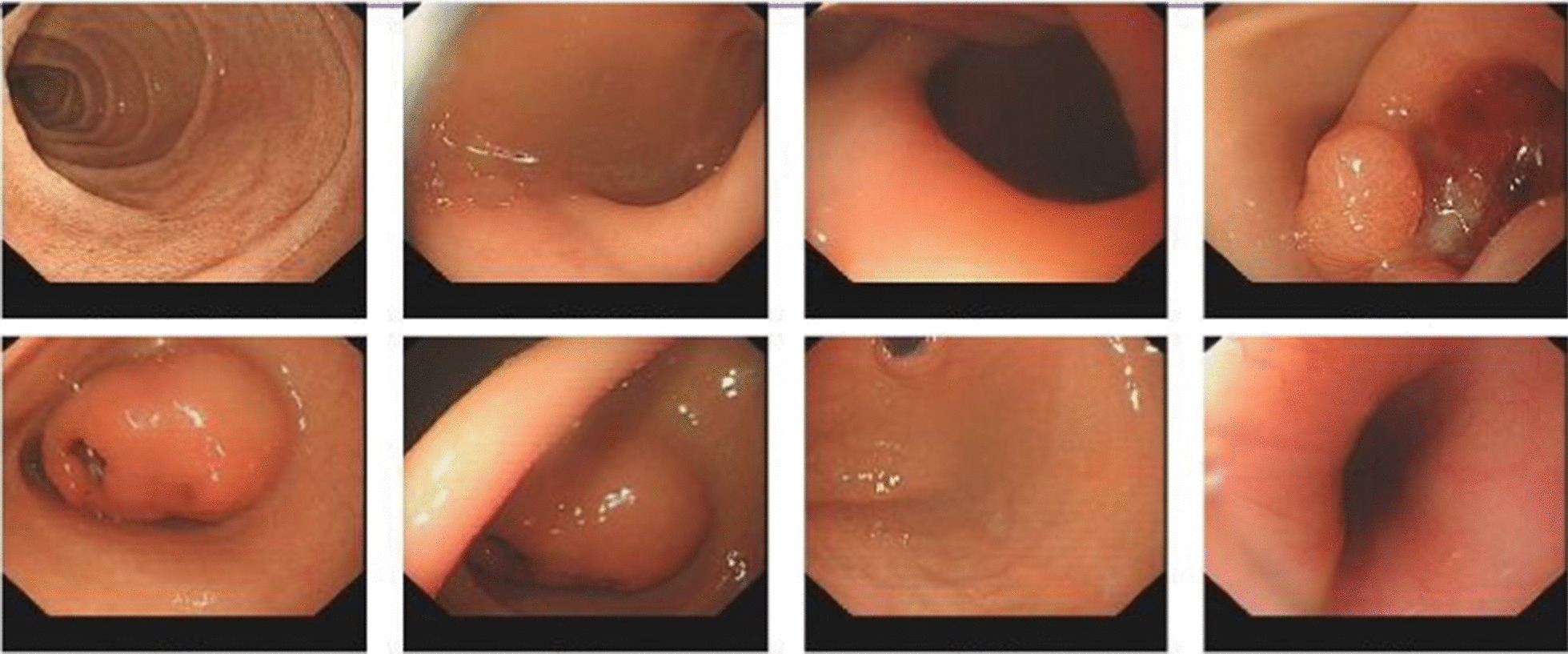
Gastroscopy shows a large lumpy mass, approximately 26 × 40 mm^2^ in size, in the antrum of the stomach, with superficial ulcers scattered on the surface of the mass, a ruptured opening, and a small amount of blood. Biopsy was performed on the mass

After admission, a series of related examinations were completed. Routine blood examination showed a red blood cell count of 1.87 × 10/L. Hemoglobin: 36 g/L; erythropoiesis: 13.3%; mean erythrocyte volume: 71.1 fL/cell; more than normal; liver function, kidney function, electrolytes, coagulation, routine urine, routine stool, and tumor tests were normal. Total abdominal plain scan and enhancement showed 1. a gastric antrum lumen mass with ulceration, size approximately 40 × 33 mm^2^, leading to consideration of a mesenchymal tumor, either (1) neuroendocrine, (2) glomus, (3) stromal, or (4) other; and 2. a small amount of pelvic fluid (Fig. 1). Gastroscopy revealed a large lumpy mass, approximately 26 × 40 mm^2^ in size, in the gastric antrum, with superficial ulcers scattered on the surface of the mass, as well as a ruptured opening and a small amount of blood. The biopsy was performed on the mass (Fig. 2). Microscopic examination of the biopsy showed that the tumor tissue was located under the mucosa. The pathological diagnosis was a "gastric antrum" spindle cell tumor. On the first day of admission, 2 units of an isotype suspension of leukocytes and erythrocytes were injected. Reexamination the next day showed a hemoglobin of 85 g/L; on the fifth day after admission, 1.5 units of an isotype suspension of leukocytes and red blood cells were injected, and the next day, hemoglobin was rechecked and measured at 105 g/L. After the exclusion of surgical contraindications, Distal gastrectomy and gastrojejunostomy were performed on the 7th day after admission, and the operation was successful.

**Fig. 3:**
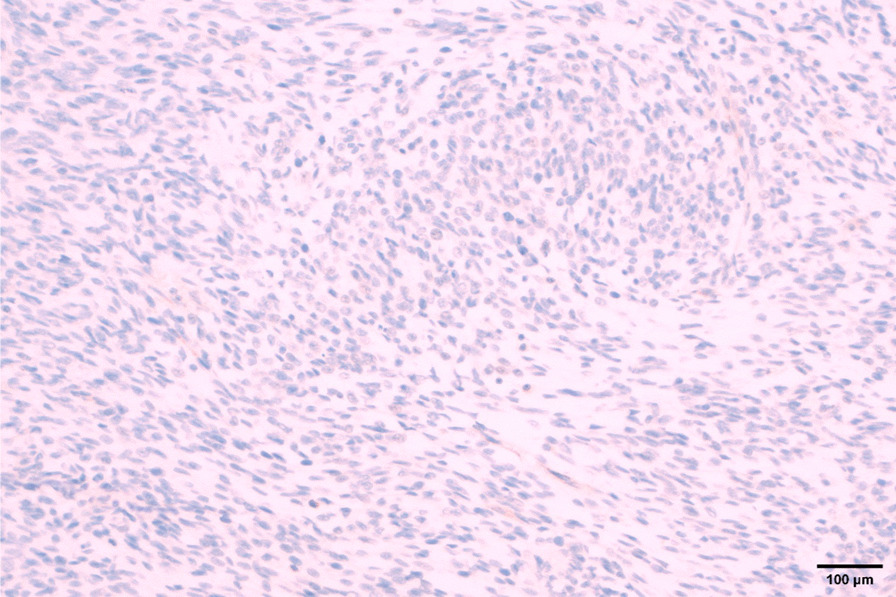
Immunostaining reveals that the mass was negative for SDHB (× 200)

**Fig. 4:**
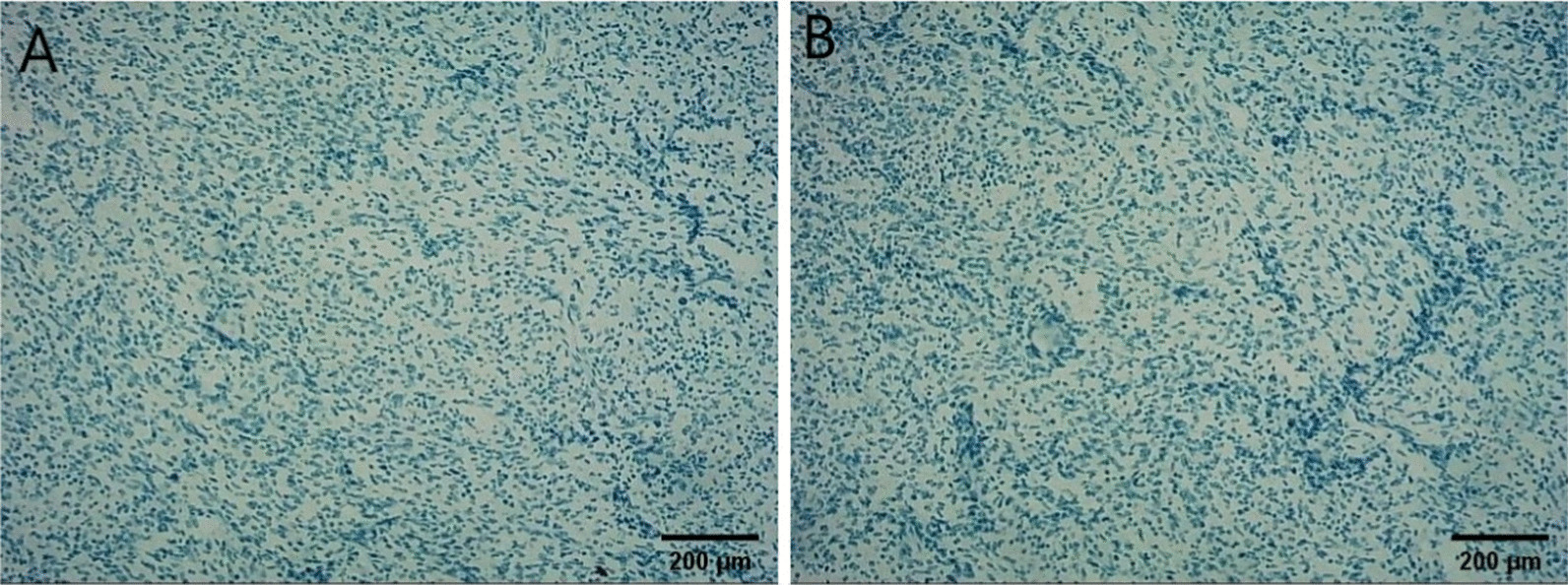
Immunohistochemistry: ALK was negative (**a**), and the negative control was normal (**b**), **a** and **b** are separated by A white line

Intraoperative exploration: no ascites in the abdominal cavity, no nodules in the liver or spleen, and no masses in the pelvic cavity, rectum, sigmoid colon, descending colon, transverse colon, ascending colon, or ileocecal area. There was a mass in the antrum of the stomach, approximately 5 × 5 cm^2^ in size, with no serous membrane accumulation. After sufficient dissection, the gastric wall was cut open, 1 cm of the gastric wall around the edge of the tumor was dissected, and the tumor was completely removed. It was found that the tumor had accumulated near the pyloric sphincter, accounting for half of the gastric circumference. The stump could not be closed by suture, so distal gastrectomy and gastrojejunostomy were decided. One week after the operation, upper gastrointestinal radiography showed changes after the distal gastrectomy, and the anastomotic stoma showed no signs of stenosis or leakage. Postoperative pathology showed that the "gastric antrum mass" was consistent with a GIST, accompanied by bleeding and cystic degeneration. The tumor was mainly located in the muscularis propria, with a maximum diameter of about 4.8 cm, a mitotic count of  > 10/5 mm^2^, and an NIH Risk Rating (2008 Modified) of high risk. No tumor was observed in "duodenal incision margin, fracture end of the suture, and gastric fracture end on the opposite side of suture". Immunohistochemical results (both positive and negative controls were set): SDHB (−) (Fig. 3), and tumor cell S-100(+), SOX10(−), CD34 (+), SMA (partial+), DOG-1 (+), CD117(+), KI-67 (positive in 20% + of the subjects and 40% + of the hotspots) (Additional file 1: e-Fig. 1, Additional file 2: e-Fig. 2, Additional file 3: e-Fig. 3, Additional file 4: e-Fig. 4, Additional file 5: e-Fig. 5, Additional file 6: e-Fig. 6, Additional file 7: e-Fig. 7), We used the Zeiss microscope, the type is Axio Scope. The image acquisition software is Democam, and the pixel is 5474 × 3648. Genetic tests showed missense mutations in ALK and TSC1, with mutation abundances of 50.14% and 49.22%, respectively. We selected lesion tissues for genetic testing with high-throughput sequencing, also known as "next-generation" sequencing technology [3]. In the testing, we again found that ALK and TSC1 had gene mutations. ALK: The 357 nucleotide G on exon 1 was replaced by T, which resulted in the substitution of amino acid glutamate (E) at 119 in the corresponding protein sequence by amino acid aspartic acid (D). The abundance of this mutation in the sample was 50.14%. TSC1: The 2281 nucleotide T located on exon 18 was replaced by nucleotide C, resulting in the substitution of amino acid tyrosine (Y) at position 761 by amino acid histidine (H) in the corresponding protein sequence. The mutation abundance in the sample was 49.22%. In addition, ALK rearrangement was detected. Since ALK fusion often occurs in intron regions, extra attention is given to this section when designing probes. The probes designed for ALK fusion are the exon region of the ALK gene and part of the intron region (the part where fusion usually occurs). In addition, we also performed immunohistochemistry for ALK on the tumor specimens, which were ALK negative (after we established a side-tumor control) (Fig. 4), We used the Olympus microscope, the type is BX53. The image acquisition software is OLYMPUS cellEens Standard, and the pixel is 640 × 480. The patient was discharged from the hospital on the ninth day after the surgery and was followed up regularly afterwards. The case report was written three months after the surgery, and the patient was generally in good condition.

## Discussion and conclusions

Generally, GISTs are dominated by KIT and PDGFRA mutations and mainly occur in adults [4]. GISTs without KIT and PDGFRA mutations are called wild-type GISTs [5]. It has been reported that 88% of patients have the succinic dehydrogenase-deficient type (66% have SDHX mutations and 22% have SDHC promoter hypermethylation) [6], while a few patients show mutations in NF1 and BRAF V600E and gene fusion of NTRK3 and FGFR1 [7–9]. The case reported here is a child with an SDH-deficient type GIST, and mutations in the ALK and TSC1 genes. A case of GIST with an PDGFRA D842V mutation in an adult was previously reported, and it was found that the patient also showed ALK expression that may be a potential therapeutic target for treating imatinib-resistant stromal tumors [10]. It has also been reported that ALK rearrangement was found in an elderly patient with a GIST in the small intestine [11], while the TSC1 mutation is associated with the occurrence of breast cancer, prostate cancer, lung cancer, colorectal cancer, esophageal cancer, and gastric cancer [12].

In this case, the patient’s GIST was SDHB(−), and missense mutations of ALK and TSC1 were observed, which have not been reported in previous studies. At the 2-month follow-up, when we wrote this report, the patient was healthy, without recurrence or metastasis. ALK tyrosine kinase inhibitors may be recommended if recurrence and metastasis are found during subsequent follow-up.

## Supplementary Information

**Additional file 1: e-Fig. 1** Immunostaining reveals that the mass was positive for S-100(× 200)

**Additional file 2 : e-Fig. 2** Immunostaining reveals that the mass was negative for SOX10(× 200)

**Additional file 3: e-Fig. 3** Immunostaining reveals that the mass was positive for CD34(× 200)

**Additional file 4: e-Fig. 4** Immunostaining reveals that the mass was partially positive forSMA (× 200)

**Additional file 5: e-Fig. 5** Immunostaining reveals that the mass was positive for DOG-1(× 200)

**Additional file 6: e-Fig. 6** Immunostaining reveals that the mass was positive for CD117(× 200)

**Additional file 7: e-Fig. 7** Immunostaining reveals that the mass was positive for KI-67(× 200) in 20% of the subjects and approximately 40% of the hotspots

## Data Availability

Not applicable.

## Abbreviations

GIST: Gastrointestinal stromal tumors
SDH: Succinic dehydrogenase
