# Supplementary figures and images for: Mutations in ALK and TSC1 in a gastrointestinal stromal tumor: a case report
Source: BMC Surg. 2021 Apr 20;21:202. doi: 10.1186/s12893-021-01208-0 (PMC8059153; doi:10.1186/s12893-021-01208-0)

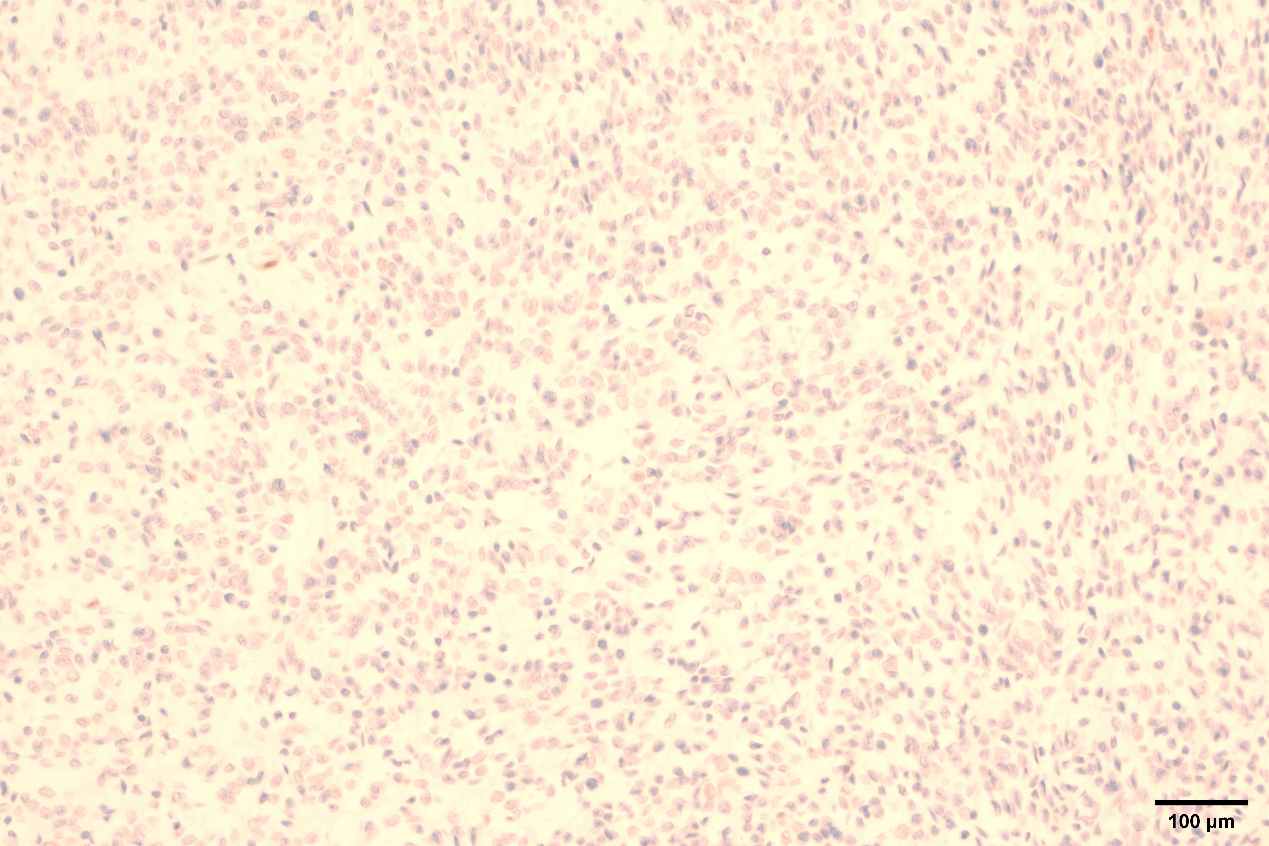


e-Fig 1

Supplement: Supplementary file 1 — Additional file 1: e-Fig. 1 Immunostaining reveals that the mass was positive for S-100(× 200) [file 12893_2021_1208_MOESM1_ESM.docx]

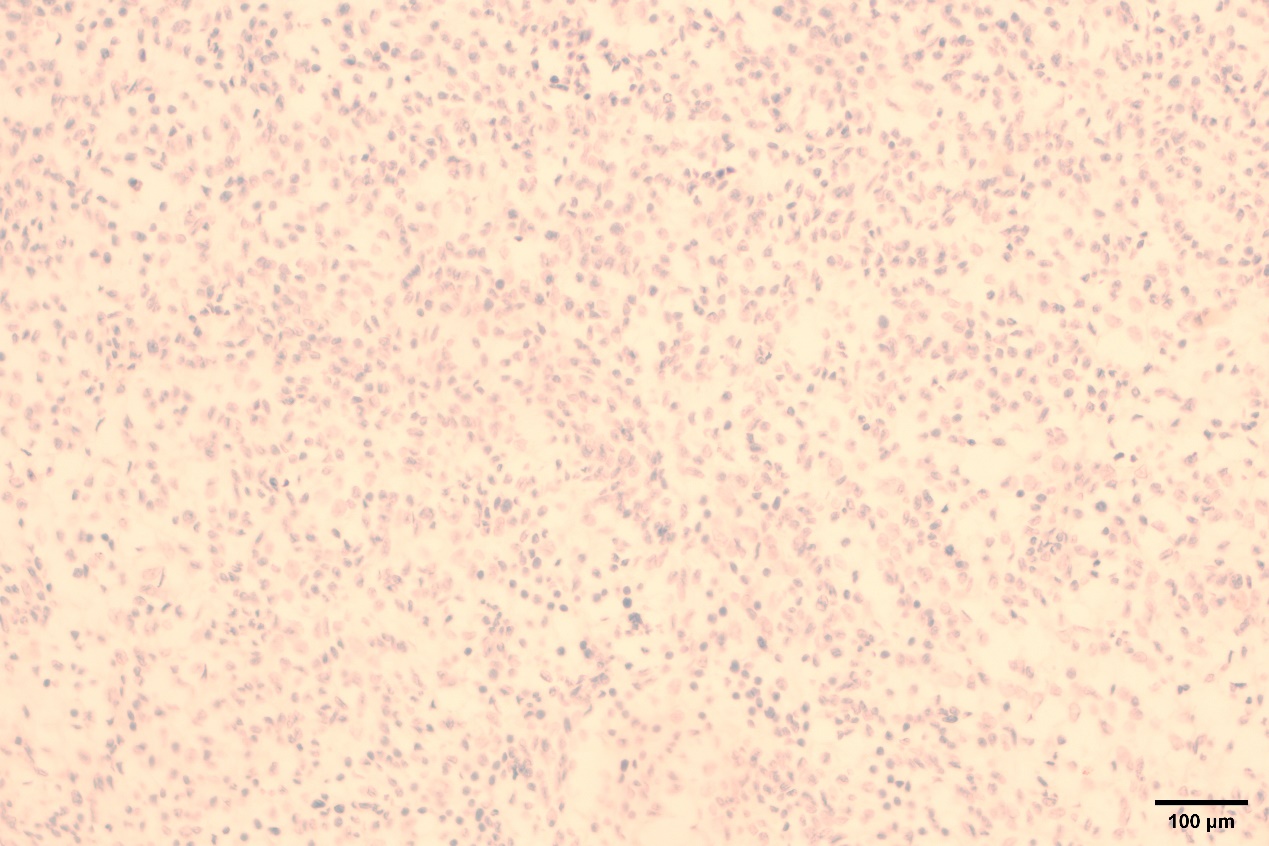


e-Fig 2

Supplement: Supplementary file 2 — Additional file 2 : e-Fig. 2 Immunostaining reveals that the mass was negative for SOX10(× 200) [file 12893_2021_1208_MOESM2_ESM.docx]

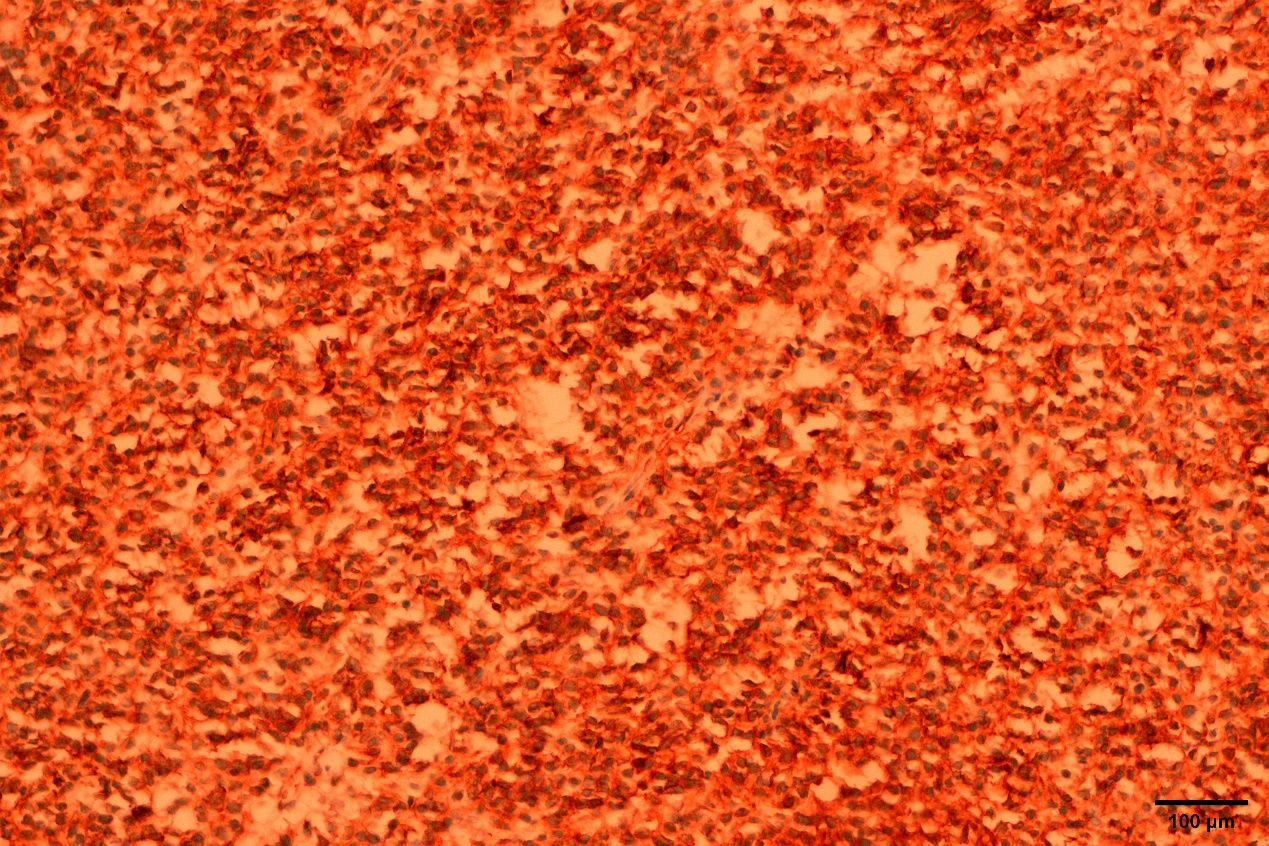


e-Fig 3

Supplement: Supplementary file 3 — Additional file 3: e-Fig. 3 Immunostaining reveals that the mass was positive for CD34(× 200) [file 12893_2021_1208_MOESM3_ESM.docx]

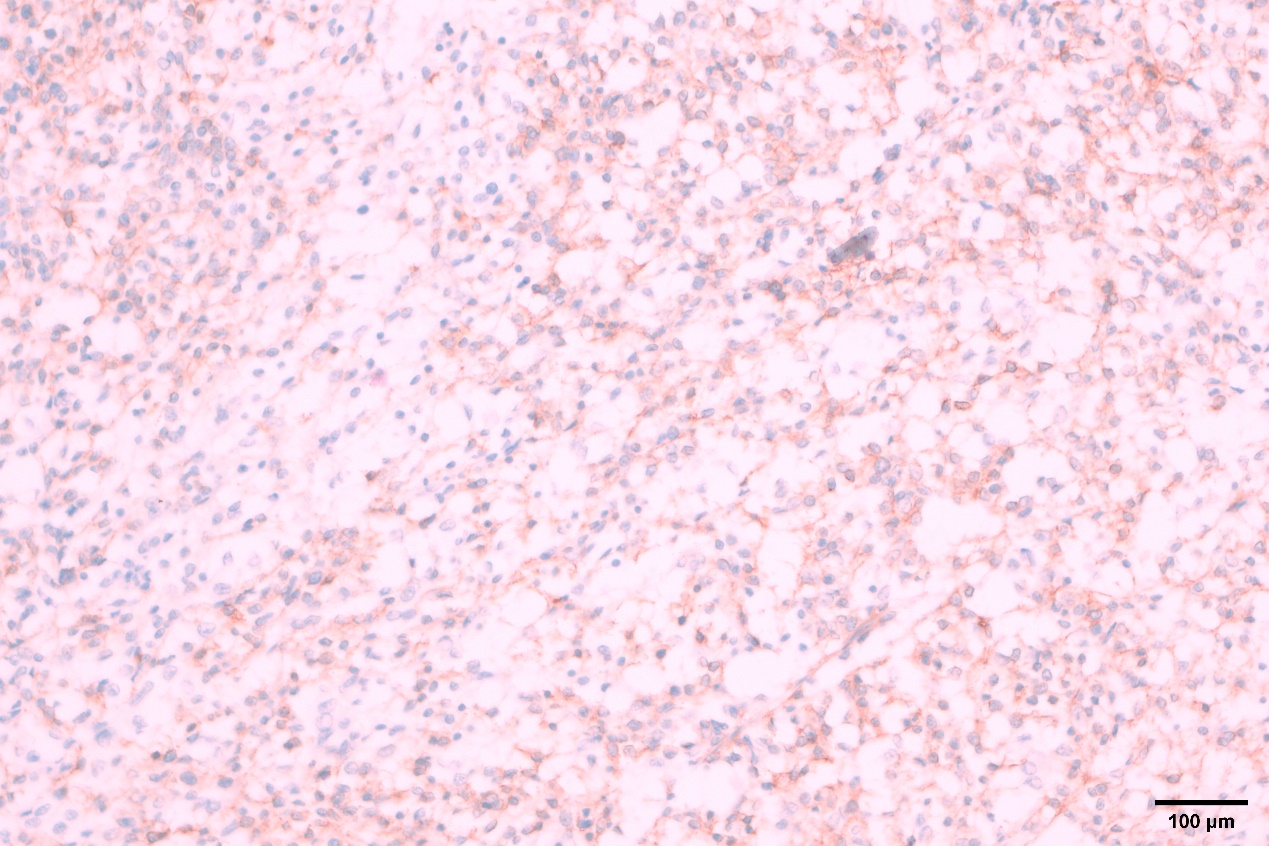


e-Fig 4

Supplement: Supplementary file 4 — Additional file 4: e-Fig. 4 Immunostaining reveals that the mass was partially positive forSMA (× 200) [file 12893_2021_1208_MOESM4_ESM.docx]

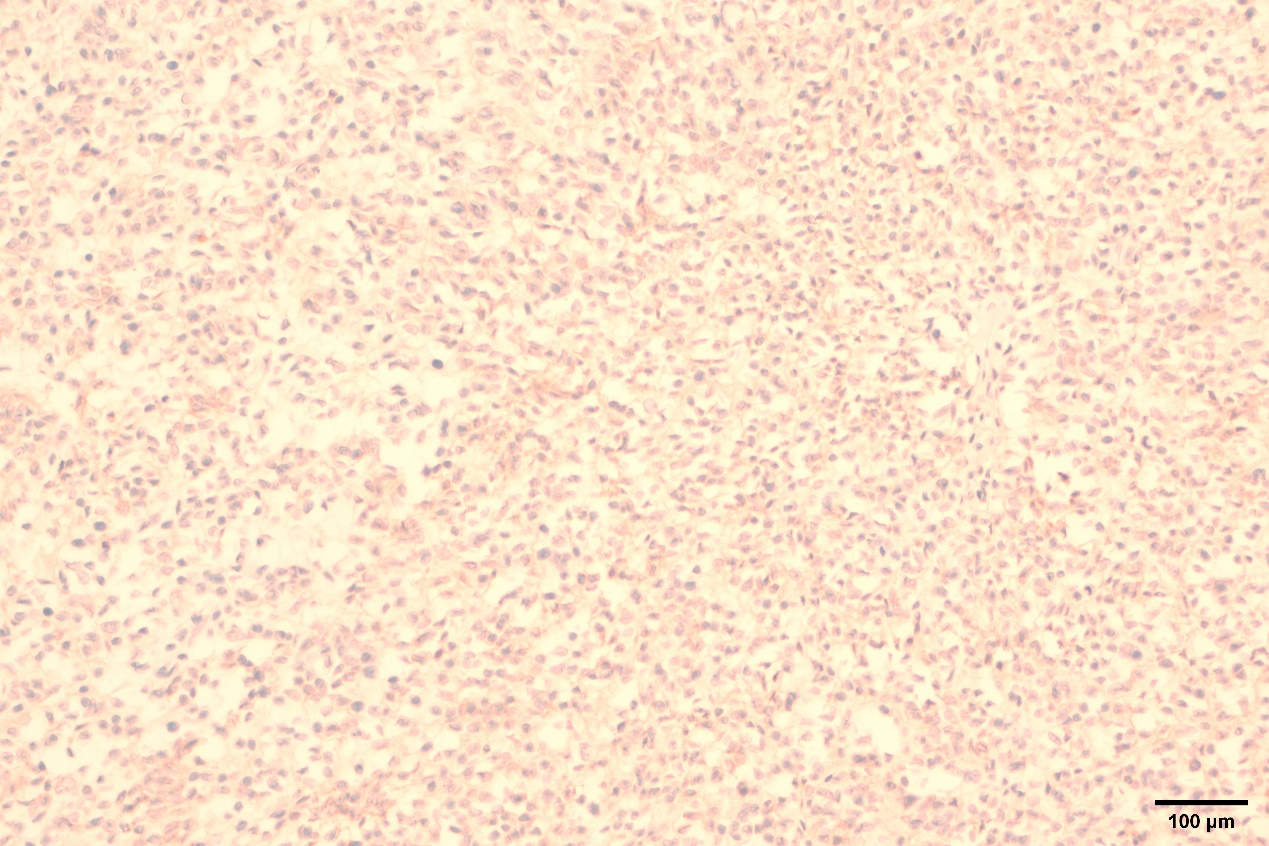


e-Fig 5

Supplement: Supplementary file 5 — Additional file 5: e-Fig. 5 Immunostaining reveals that the mass was positive for DOG-1(× 200) [file 12893_2021_1208_MOESM5_ESM.docx]

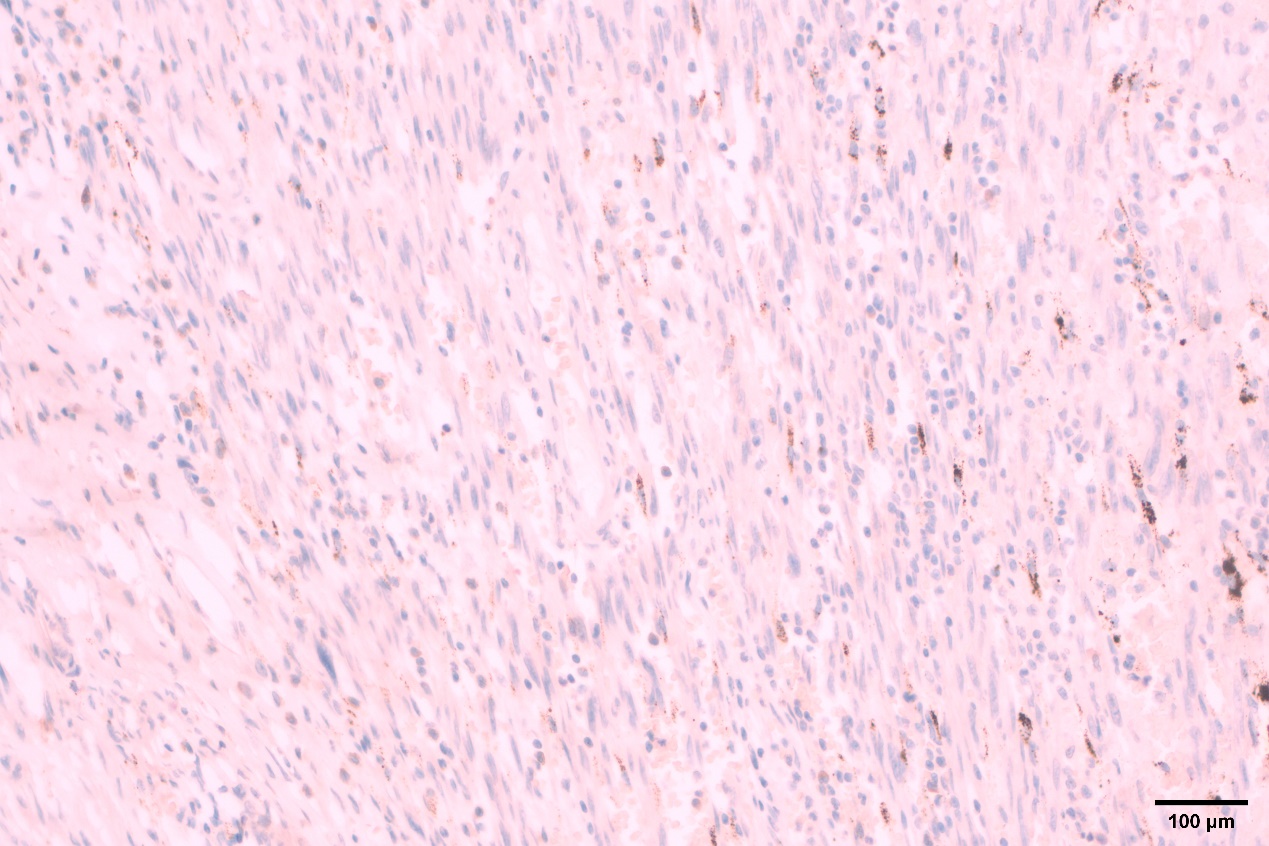


e-Fig 6

Supplement: Supplementary file 6 — Additional file 6: e-Fig. 6 Immunostaining reveals that the mass was positive for CD117(× 200) [file 12893_2021_1208_MOESM6_ESM.docx]

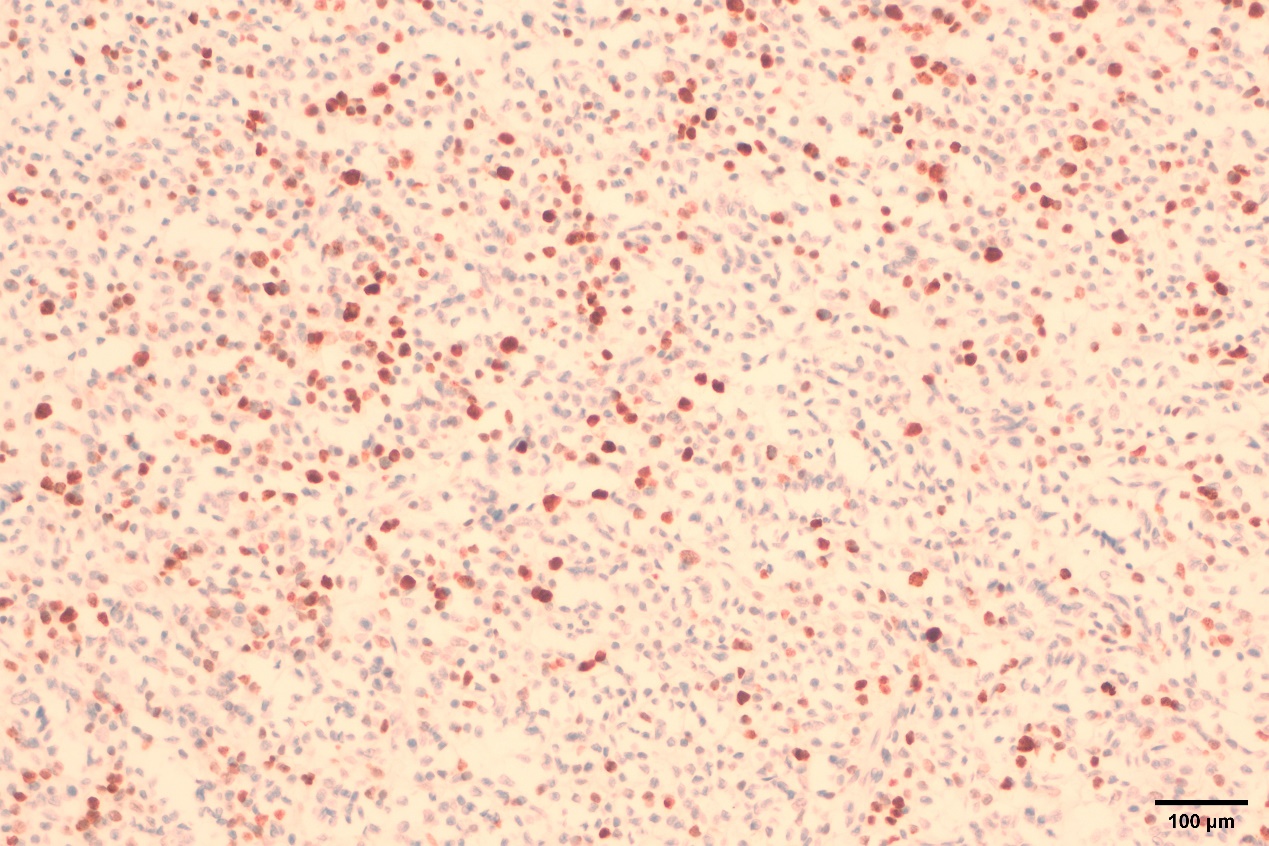


e-Fig 7

Supplement: Supplementary file 7 — Additional file 7: e-Fig. 7 Immunostaining reveals that the mass was positive for KI-67(× 200) in 20% of the subjects and approximately 40% of the hotspots [file 12893_2021_1208_MOESM7_ESM.docx]
